# Exudative Retinal Detachment due to Coats Disease in a Teenager with Senior-Loken Syndrome: Case Report and Review of Literature

**DOI:** 10.7759/cureus.4460

**Published:** 2019-04-15

**Authors:** Kamalul Khusus Khairil-Ridzwan, Adnan Azian, Hashim Hanizasurana, Ismail Shatriah

**Affiliations:** 1 Ophthalmology, Hospital Selayang, Batu Caves, MYS; 2 Ophthalmology, School of Medical Sciences, Universiti Sains Malaysia, Kubang Kerian, MYS

**Keywords:** senior-loken syndrome, retinal dystrophy, coats disease, exudative retinal detachment

## Abstract

Senior-Loken syndrome is a rare disorder that presents in the first two decades of life. It commonly manifests with nephronophthisis and retinal dystrophy. We describe a teenager who had end-stage renal failure presenting with bilateral visual impairment due to retinal dystrophy with concomitant unilateral Coats disease and exudative retinal detachment. The patient was treated with a combination of endolaser photocoagulation and external drainage of the subretinal fluid. The final visual acuity remained poor in both eyes. Options of treatment in this challenging situation is discussed in this case report.

## Introduction

Senior-Loken syndrome is a rare autosomal recessive hereditary syndrome characterized by renal and retinal disorders. It was first described in 1961 by Senior et al. and Loken et al. [1-2]. It is present in approximately 10% to 15% of childhood genetic renal disease known as nephronophthisis [3]. This group of disease affects about one in 50,000 births. The initial presenting symptoms of the renal disease are polyuria and polydipsia. The onset is gradual and most cases do not manifest until the renal failure is advanced. There is a spectrum of associated manifestations, including skeletal, dermatological, and cerebellar anomalies [4].

Here we report a case of Senior-Loken syndrome presenting with an exudative retinal detachment secondary to Coats disease. We discuss possible treatment options and the visual outcome in this uncommon ocular problem in teenage patients.

## Case presentation

A 14-year-old Chinese boy presented with a complaint of progressive worsening vision in both eyes for two years. The patient had been aware of poor vision since childhood, and there had recently been further deterioration. His mother noticed that he had poor eye contact since the age of four months. Both parents consulted an ophthalmologist once when the patient was nine years old. They were informed of poor visual prognosis, and declined ophthalmology follow-up since then.

Past medical history revealed that the patient developed lethargy and severe vomiting three years earlier, and underwent a thorough systemic examination and work-up. Ocular examination at that time confirmed bilateral optic atrophy and pigmentary retinal changes. Abdomen ultrasonography showed small bilateral renal cysts and coarse liver texture. No liver cysts were observed. He was diagnosed with end-stage renal failure, anemia, and hypertension. He was started on continuous cycling peritoneal dialysis. Subsequently, the chromosomal studies confirmed 46XY. The diagnosed was revised. His clinical manifestation was consistent with Senior-Loken syndrome. The patient’s general condition was stable and he was compliant with treatment.

On examination, the patient was a small build teenager with a height of 140 cm and a weight of 33.8 kg. His blood pressure was in the normal range on medication. There was no evidence of abnormal sexual development or spinal deformity.

**Figure 1:**
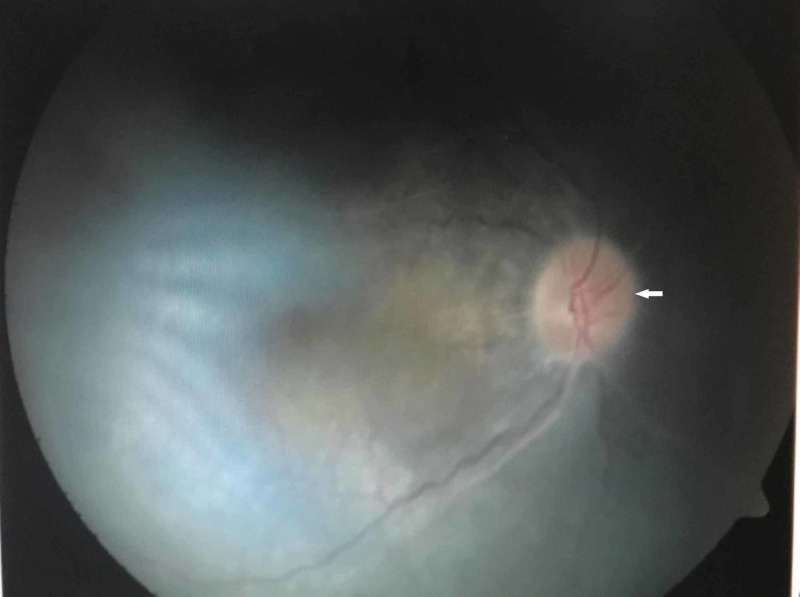
Funduscopy examination showing pale optic discs (white arrow)

**Figure 2:**
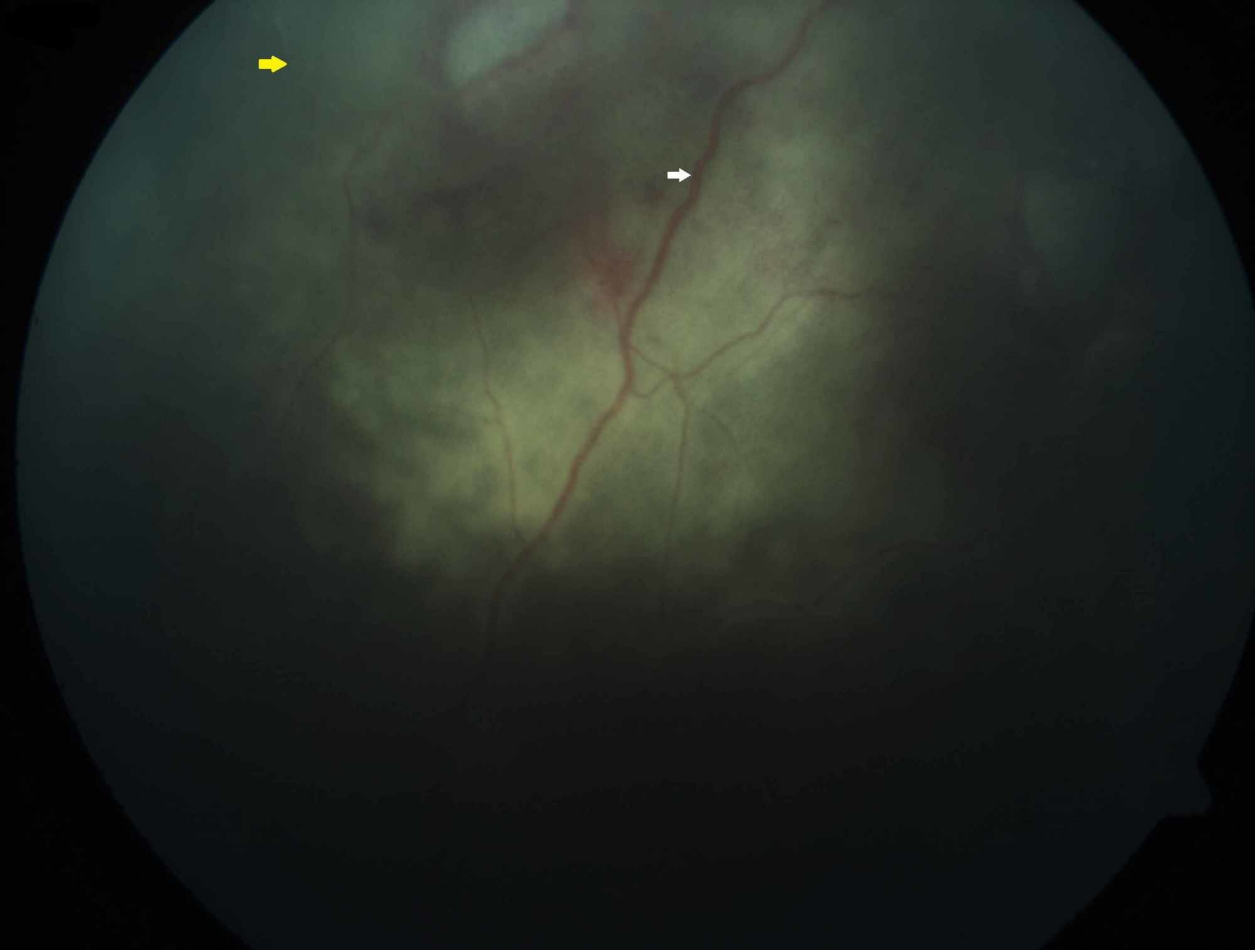
Funduscopy examination showing sclerosed (yellow arrow) and attenuated retina vessels (white arrow)

The visual acuity was counting fingers at one foot in both eyes. He had nystagmus bilaterally. Slit lamp examination showed moderate nucleus sclerosis in both eyes. Funduscopy revealed bilateral pale optic discs, hypopigmentation at the mid-periphery of the retina, and sclerosis with attenuated vessels at all quadrants of the retina (Figures 1-2).

**Figure 3:**
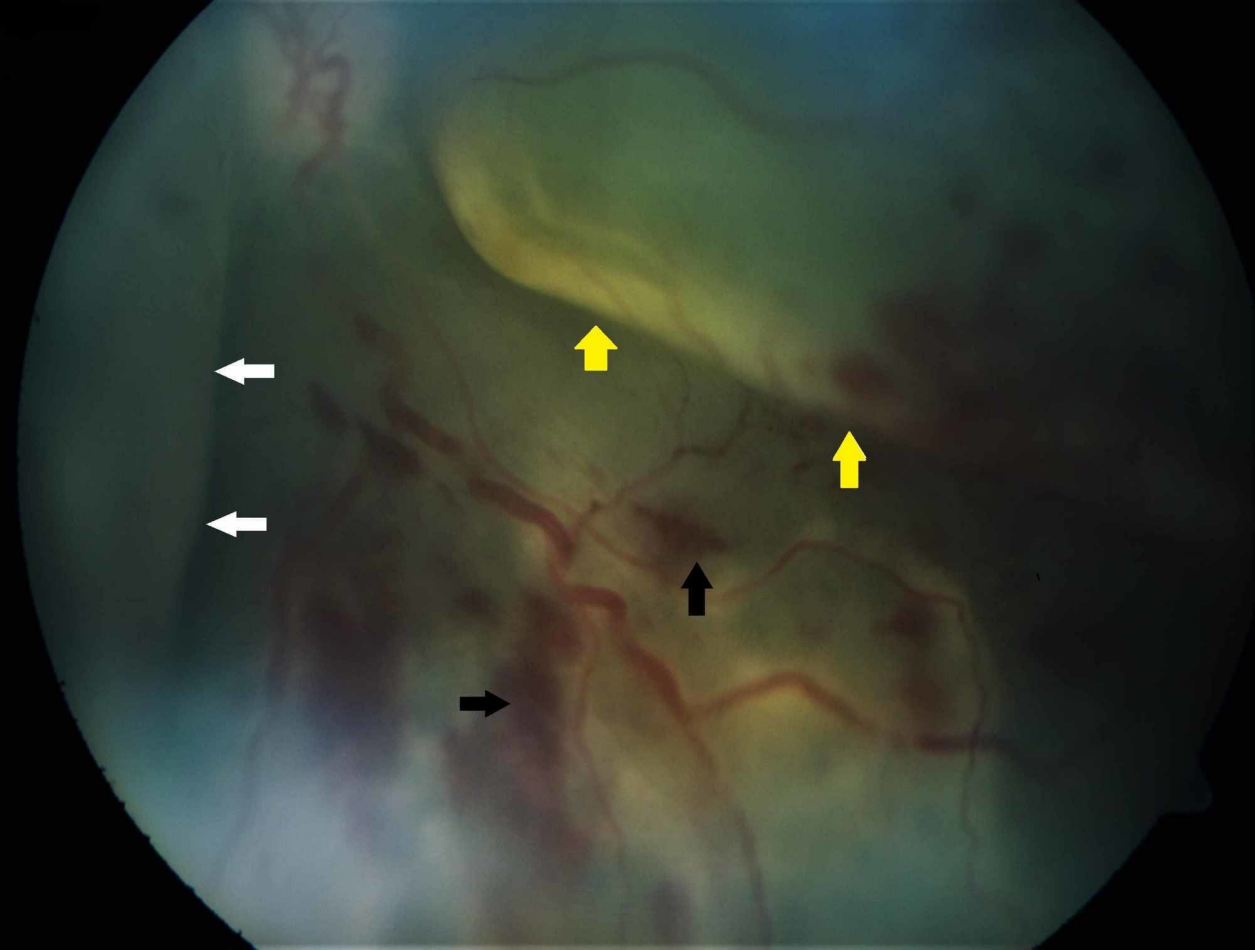
Funduscopy examination showing telangiectatic vessels at the periphery with retinal hemorrhages (black arrows), subretinal exudates (white arrows), and exudative retinal detachment (yellow arrows)

In the left fundus, there were telangiectatic vessels at the periphery, retinal hemorrhages, and subretinal exudates with a shallow exudative retinal detachment (Figure 3).

Fundus fluorescence angiography was deferred in view of his renal condition. Optical coherence tomography revealed foveal atrophy in the right eye. However, the images were poor in both eyes due to nystagmus.

The patient underwent one session of laser photocoagulation under general anesthesia in the left eye. The condition persisted, and an external drainage of the subretinal fluid was performed one month later. His best-corrected visual acuity was hand movement, and the retina appeared flat. The collection of subretinal fluid resolved after the procedure.

The patient was monitored closely during post-operative periods. The visual acuity in both eyes at one year postoperatively remained hand movement. Both retinae were flat.

## Discussion

Senior-Loken syndrome presents in the first two decades of life and causes a tapetoretinal degeneration with a variable range of retinal lesions. It ranges from severe Leber amaurosis to a more typical retinitis pigmentosa. Other ocular manifestations include cataract, spherophakia, and anterior lenticonus [5].

Coats disease is a rare unilateral eye disorder with male predominance that occurs in the second and third decades. It is an idiopathic retinal telangiectasia that is associated with subretinal exudation, and it frequently results in exudative retinal detachment. The association of Coats disease with Senior-Loken syndrome is rare, with only few reported cases in the literature [5-7].

Table 1 summarizes published pediatric cases of Senior-Loken syndrome with Coats disease and includes our patient [5-7]. There were three male and one female patient. The youngest was nine years old, and the oldest was 15 years old. All patients presented with advanced bilateral retinitis pigmentosa (or retinitis-pigmentosa like disease) and unilateral exudative retinal detachment due to Coats disease.

**Table 1:** Published case reports of Senior-Loken syndrome with Coats disease (1985-2019) OD: oculus dexter (right eye); OS: oculus sinister (left eye); OU: oculus uterque (both eyes).

Author/ Year	Age / Gender	Visual acuity on presentation	Ocular Diagnosis	Complication	Treatment	Final visual acuity
Schuman et al./ 1985 [5]	15 / Male	OD: Perception of light	OU: Retinitis pigmentosa, OD: Coats disease and exudative retinal detachment	OD: Neovascular glaucoma with subretinal exudate and haemorrhage	Enucleation	OD: No perception of light
Oyama et al./2004 [6]	14 / Male	OU: Poor visual acuity	OU: Retinitis pigmentosa, Keratoconus and cataract, OS: Coats disease and exudative retinal detachment	OS: Neovascular glaucoma	OU: Vitrectomy and endolaser photocoagulation, OS: cyclophotocoagulation	OU: No perception of light
Sato et al. / 2007 [7]	9 / Female	OD: 30/100 OS: 30/100	OU: Retinitis pigmentosa, Coats disease and total exudative retinal detachment	OD: Secondary glaucoma, OS: Neovascular glaucoma	OU: Vitrectomy, OD: Cryotherapy, cyclophotocoagulation	OU: No perception of light
Khairil-Ridzwan et al./ 2019	14 / Male	OU: Counting fingers one foot	OU: Retinitis pigmentosa, OS: Coat disease with exudative retinal detachment	Nil	OS: Endolaser photocoagulation and external drainage of subretinal fluid	OU: Hand movement

Diathermy, laser photocoagulation, cryotherapy, subretinal fluid drainage, scleral buckling, pars plana vitrectomy, and intravitreal anti-vascular endothelial growth factor therapy are known options of treatment in Coats disease. The aim of treatment is to destroy the abnormal vasculature.

Sato et al., Oyama et al., and Schuman et al. reported that their patients were treated with pars plana vitrectomy and endolaser photocoagulation/cryotherapy [5-7]. These cases were complicated by neovascular glaucoma. Cai et al. described the use of endolaser treatment using a two-port non-vitrectomy approach in 24 pediatric patients with stage 3 Coats disease with shallow retinal detachment [8]. Reattachment of the retina was seen in 96% of their patients.

Our patient was treated successfully with endolaser photocoagulation and external drainage of subretinal fluid. The aim of subretinal fluid drainage is to reattach the retina. Long-standing retinal detachment can lead to phthisi bulbi, ocular hypotony, and neovascular glaucoma. The options for drainage of subretinal fluid are divided into internal or external approaches. External subretinal drainage is preferred in cases of peripheral bullous retinal detachment. 

Rishi et al. reported treatment outcomes in 307 Indian eyes with Coats disease [9]. Exudative retinal detachment was present in 51.2%. Subretinal drainage was performed in 21 cases (6.8%), and retina reattachment was observed in 12 cases. Vitrectomy was performed in eight cases (4.2%) and attached retina was found in seven cases. Cryotherapy was performed in 17 cases (7.8%), and reattached retina was noted in 11 cases. Eighteen cases (9.8%) had scleral buckling, and only six cases had reattachment of the retina. Other patients (80 cases, 33.3%) had laser photocoagulation treatment and reattached retina was observed in 56 cases [9].

Recently, injection of anti-vascular endothelial growth factor has become one of the preferred choices for treating Coats disease with exudative retinal detachment. The aims are to reduce trauma and minimize treatment intervention. The procedure is indicated in patients with stage 3b and worse. It is usually used as an adjunct to other treatment options in Coats disease such as laser photocoagulation, cryotherapy, subretinal drainage and vitrectomy, and not as a monotherapy.

Parlak and Saatci reviewed the use of bevacizumab, ranibizumab, and pegaptanib as an adjuvant treatment with subsequent necessary treatments as mentioned above [10]. There were no reports on the use of aflibercept in children with Coats disease. Resolution of subretinal fluid and exudation, and regression of telangiectasia had been documented by those patients reviewed by Parlak and Saatci [10].

The final visual acuity was reported as poor in those cases [5-7]. Schuman et al. reported that their patient ultimately underwent enucleation due to painful neovascular glaucoma. Sato et al. and Oyama et al. highlighted that their patients were blind with loss of light perception in the affected eye. The current visual acuity in our patient is hand movement in both eyes. We are monitoring him closely during follow-up visits, and no complications have been observed. His current visual acuity is poor and consistent with underlying retinal dystrophy in his condition.

## Conclusions

Pediatric patients with renal failure require mandatory ophthalmology assessment. Senior-Loken syndrome with exudative retinal detachment due to Coats disease is rare. A combination of endolaser photocoagulation and subretinal fluid drainage was effective in our patient. More reports are required to assist ophthalmologists in managing this rare situation.
